# Lipopolysaccharide Specific Immunochromatography Based Lateral Flow Assay for Serogroup Specific Diagnosis of Leptospirosis in India

**DOI:** 10.1371/journal.pone.0137130

**Published:** 2015-09-04

**Authors:** Shanmugam Vanithamani, Santhanam Shanmughapriya, Ramasamy Narayanan, Veerapandian Raja, Murugesan Kanagavel, Karikalacholan Sivasankari, Kalimuthusamy Natarajaseenivasan

**Affiliations:** Medical Microbiology Laboratory, Department of Microbiology, Centre of Excellence in Life Sciences, Bharathidasan University, Tiruchirappalli, 620 024, India; University of Parma, ITALY

## Abstract

**Background:**

Leptospirosis is a re-emerging infectious disease that is under-recognized due to low-sensitivity and cumbersome serological tests. MAT is the gold standard test and it is the only serogroup specific test used till date. Rapid reliable alternative serogroup specific tests are needed for surveillance studies to identify locally circulating serogroups in the study area.

**Methods/Principal Findings:**

In the present investigation the serological specificity of leptospiral lipopolysaccharides (LPS) was evaluated by enzyme linked immunosorbent assay (ELISA), dot blot assay and rapid immunochromatography based lateral flow assay (ICG-LFA). Sera samples from 120 MAT positive cases, 174 cases with febrile illness other than leptospirosis, and 121 seronegative healthy controls were evaluated for the diagnostic sensitivity and specificity of the developed assays. LPS was extracted from five locally predominant circulating serogroups including: Australis (27.5%), Autumnalis (11.7%), Ballum (25.8%), Grippotyphosa (12.5%), Pomona (10%) and were used as antigens in the diagnostics to detect IgM antibodies in patients’ sera. The sensitivity observed by IgM ELISA and dot blot assay using various leptospiral LPS was >90% for homologous sera. Except for Ballum LPS, no other LPS showed cross-reactivity to heterologous sera. An attempt was made to develop LPS based ICG-LFA for rapid and sensitive serogroup specific diagnostics of leptospirosis. The developed ICG-LFA showed sensitivity in the range between 93 and 100% for homologous sera. The Wilcoxon analysis showed LPS based ICG-LFA did not differ significantly from the gold standard MAT (P>0.05).

**Conclusion:**

The application of single array of LPS for serogroup specific diagnosis is first of its kind. The developed assay could potentially be evaluated and employed for as MAT alternative.

## Introduction

Leptospirosis is an important reemerging zoonotic disease with worldwide distribution [1]. More than 250 pathogenic *Leptospira* serovars are known to infect humans [2]. The leptospires colonize the internal organs and isolation of leptospires from infected host is possible from lungs, liver and kidneys [3]. The symptoms of leptospirosis vary from mild flu like illness to multi organ failure and in advanced condition leads to death of the infected host [4]. The clinical presentation is difficult to distinguish leptospirosis from dengue, malaria, influenza and many other febrile diseases [5]. The incidence, mortality rates and an increasing number of outbreaks identified leptospirosis as a fatal disease worldwide [6].

Performing microscopic agglutination test (MAT) to demonstrate fourfold raise in antibody titre and isolation of leptospires from the infected specimen are the direct confirmatory evidence of leptospirosis [7]. Although MAT is considered as a gold standard test for diagnosis of leptospirosis, it is laborious, requires a large panel of live leptospiral culture and needs expertise for interpretation of results [8]. As leptospirosis is endemic in developing or underdeveloped countries, there is an urgent need for rapid, reliable and inexpensive diagnostic formats. Alternative diagnostic formats including, Enzyme linked immunosorbent assay (ELISA), dipstick assay, macroscopic agglutination test, microcapsule agglutination has been developed several years ago [9]. Of these methods, ELISA offers reasonable sensitivity and the possibility of handling many samples at a time and has been developed and evaluated with number of modifications starting with the use of crude antigens to conserved peptides [10]. But the currently evaluated diagnostic formats are mainly based on the conserved protein antigens and cannot be employed for serogroup level identification. Till date there is no serogroup specific diagnostics to replace MAT.

LPS present in the outer membrane of *Leptospira* is one of the major essential immunodominant antigens. The antigenic potential of LPS initiates the pathogenesis and helps in the serogroup level identification of *Leptospira* species [11]. In this regard, validation of such serogroup specific antigens in diagnostics can serve as an alternative for MAT.

In order to develop a serogroup specific diagnosis of leptospirosis, leptospiral LPS was extracted from the predominant locally circulating serovars and evaluated in the form of antigen array in different diagnostic formats. Moreover in the study, we tried to develop an Immunochromatography—Lateral Flow Assay (ICG-LFA) for IgM antibody detection. The developed ICG-LFA was evaluated for its sensitivity and specificity to find its application for serogroup specific diagnosis in endemic regions like India.

## Materials and Methods

The standards for the Reporting of Diagnostic accuracy testing (STARD) were followed during the conduct of this study [12].

### Patients, Case Definition and Ethics

Through our hospital based surveillance between October 2013 and December 2014 in Chennai, Tamilnadu, India, sera samples from a total of 320 clinically suspected (patients hospitalized with clinical manifestations including fever, body ache, myalgia, icterus, conjunctival suffusion, arthritis, rigors, pain of the abdomen, breathlessness, subconjunctival haemorrhages, and jaundice with acute renal failure) leptospirosis cases were collected. The samples were collected during the early phase of illness (0 to 10 days after onset of the disease). Of the 320 suspected cases, 200 had a laboratory confirmed diagnosis for leptospirosis (isolation of leptospires from blood or urine, seroconversion: negative to a titre of 1:160 or more or four-fold rise in titre, IgM-ELISA with a titre of ≥1:160 using heat extracted antigen) and remaining 120 were considered discarded for leptospirosis and hence not included in the study (S1 Table). Of the 200 laboratory confirmed cases, 120 sera samples had a MAT titer of ≥1:160 (closest titre to 1:200, which is the ideal cut-off in endemic areas) [13;14] and thus included in the study. A total of 121 seronegative healthy controls selected from the neighborhood of the cases matching for age (±5 yrs) and sex, and 174 patients who were hospitalized for febrile diseases other than leptospirosis were also included to study the specificity of the developed diagnostics (Table 1). Informed written consent was obtained from both cases and controls before blood sampling, and the study protocol was approved by the Institutional Ethics Committee (IEC) of Bharathidasan University (DM/2007/101/373/2) as well as permitted by the Directorate of Health Services (Ref. No. 5796/ TV 1/07), Government of Tamilnadu. The obtained sera samples were stored at-80°C until use.

**Table 1:** Case definition and groupings of the patients included in the study.

Group	Description	Number of cases
A	Clinically suspected and laboratory confirmed leptospirosis*	200
B	Clinically suspected and laboratory negative leptospirosis**	120
C	Other febrile illness	
	Typhoid	37
	Malaria	44
	Dengue	45
	Hepatitis	48
D	Seronegative healthy controls	121

* The number of cases confirmed by diagnostic criteria of leptospirosis (Refer materials and methods)

** Clinically suspected and serologically negative. The study group was not included in the study.

### Homologous and Heterologous Sera

The sera samples were divided as homologous sera (HS) and heterologous sera (HES) based on the MAT positivity. Homologous sera are defined as patients’ sera that reacted with a specific serogroup in MAT and tested against LPS of the same serogroup. Heterologous sera are defined as patients’ sera that reacted with a specific serogroup in MAT and tested against LPS of other serogroups.

### Leptospiral Strains and MAT

The MAT was performed to evaluate serological evidence of leptospiral infection [8]. A panel of 12 reference strains were used which included the following serogroups: Australis (serovar Australis, strain Ballico), Autumnalis (serovar Autumnalis, strain Akiyami A), Ballum (serovar Ballum, strain Mus 127), Bataviae (serovar Bataviae, strain Swart), Canicola (serovar Canicola, strain Hond Utrecht IV), Icterohaemorrhagiae (serovar Icterohaemorrhagiae, strain RGA), Grippotyphosa (serovar Grippotyphosa, strain Moskva V), Hebdomadis (serovar Hebdomadis, strain Hebdomadis), Javanica (serovar Poi, strain Poi), Pomona (serovar Pomona, strain Pomona), Sejroe (serovar Hardjo, strain Hardjoprajitno) Pyrogenes (serovar Pyrogenes, strain Salinem). The strains were obtained from the WHO Reference Centre for Leptospirosis, Regional Medical Research Centre, ICMR, Port Blair and maintained by regular sub-culturing in Ellinghausen-McCullough-Johnson-Harris (EMJH) bovine serum albumin-Tween 80 medium (Difco Laboratories, USA) at the Medical Microbiology Laboratory, Bharathidasan University, Tiruchirapalli, Tamilnadu.

### Extraction of Leptosiral LPS and Heat Extracted Antigen

*Leptospira* were grown in liquid EMJH medium at 30°C and collected at a density of ~ 5 ×10^8^ bacteria/mL. Leptospiral LPS and LPS from other bacteria including *Serratia marcescens* and *Citrobacter freundi* were extracted following the standard hot phenol-water method [15]. The phenol phase that contained the LPS was used for further purification. The phenol phase was dialyzed extensively against water and insoluble material removed by centrifugation. LPS was prepared from five locally predominant leptospiral serogroups Autumnalis, Australis, Ballum, Grippotyphosa and Pomona. Non-pathogenic serogroup Andamana was also included for comparison. The extracted LPS were quantified by the phenol/sulfuric acid method using sucrose as a standard. *E*. *coli* O111:B4 LPS (Sigma Adrich, USA) and other bacterial LPS were used as controls. Leptospiral heat extracted antigen was prepared from 7 days old well-grown leptospiral culture (1–2x10^8^/ml) for the utilization in IgM-ELISA [16]. Protein concentrations were determined by bicinchoninic acid (BCA) method (Sigma Aldrich, USA).

### SDS-PAGE and Immunoblotting

SDS-PAGE was performed on a 12% polyacrylamide gel using a discontinuous buffer system. The extracted LPS were mixed with 2X SDS-PAGE sample loading buffer (BioRad, Hercules, CA, USA) and boiled for 5 min before loading. Electrophoresis was carried out in a vertical electrophoretic minicell unit (BIORAD, Hercules, CA, USA) for 2 h at 120V in Tris-glycine running buffer (25mM Tris, 192 mM glycine, 0.1% SDS, pH 8.3). The separated LPS were transferred to nitrocellulose membranes (0.2 μm pores size; Schleicher and Schuell, Keene, NH) [17] and blocked with 4% non-fat dry milk in Tris-buffered saline (20mM Tris, 150mM NaCl, 0.05% Tween 20, pH 7.5;TBST). Membranes were incubated with serum (1:200) from patients positive for leptospirosis, followed by incubation with anti-human IgG antibody conjugated with horseradish peroxidase (Sigma, St. Louis, Mo) and bands visualized by using 4-chloro-α- naphthol (Sigma, St. Louis, Mo).

### Enzyme Linked Immunosorbent Assay (ELISA)

ELISA was carried out using extracted LPS as described previously [18]. Checkerboard titrations were performed to determine the optimal concentrations of LPS for IgM ELISA. Two microgram of extracted LPS were coated on flat-bottom polystyrene microtiter plates (Nunc Nalgene, USA) at 4°C overnight, using carbonate coating buffer (pH 9.6), followed by blocking with 4% nonfat dry milk. Patients’ sera (1:200) in triplicate were added and incubated for 1 h at 37°C. Bound IgM was detected using ALP-conjugated anti-human IgM (Sigma-Aldrich, St. Louis, MO) at a dilution of 1:4000. Plates were developed with PNPP (4- Nitrophenyl phosphate bis [cyclo hexyl ammonium salt]) (Sigma-Aldrich, St. Louis, MO). The reaction was stopped with the addition of 50 μL of 1 N H_2_SO_4_, and the optical density was measured at 490 nm using ELISA reader (BioRad, Hercules, CA, USA).

### Dot Blot with an Array of LPS

Dot blot assay was carried out with extracted LPS of various leptospiral serogroups. The optimal LPS concentration for IgM based detection was determined as 1.5 μg (S1 Fig). Antigens were dotted on the nitrocellulose membrane and air dried. The membrane was blocked with 4% non-fat milk and incubated at 37°C for 1 hour and washed four times with PBS-T. The membrane was incubated with patients’ sera diluted in PBS-T (1: 200) for 1 hour at 37°C. The membrane was incubated with anti-human ALP conjugated-IgM (1:2000; Sigma Aldrich, USA) at 37°C for 1 hour and washed with PBS-T. The membrane was further developed with chromogenic substrate 5-bromo-4-chloro-3-indolyl phosphate Nitro-blue tetrazolium (Sigma Aldrich, USA) and the intensity was quantified densitometrically and expressed as arbitrary units (AU).

### Immunochromatography Based Lateral Flow Assay (ICG-LFA)

Fifty micrograms of extracted LPS were placed at equal distance as an array on the test (T) area and 2 μg of anti-human IgM (Sigma Aldrich, USA) was placed as an internal control (IC). The membrane was dried in a desiccator for 3 hours at 37°C. The unconjugated regions were blocked in 10 mM phosphate buffer (pH 7.2) containing 1% non-fat milk for 1 hour, washed twice with the same buffer and dried at room temperature. Glass fiber conjugate pads (GE healthcare) were dipped in gold-conjugated protein A dissolved in 2mM borate buffer (pH 7.2) supplemented with 5% sucrose. The pads were then air dried at 37°C for 2 h. Cellulose fiber sample pads (BioRad, Hercules, CA, USA) were dipped in sample pad buffer (50 mM borate buffer (pH 7.2), 5% sucrose, 0.5% Tween 20, 5% dextran, and 0.1% skim milk) and dried at 50°C [19]. Fifty microliters of patient sera was placed on the sample pad and incubated for 10 minutes. Positivity was determined by the development of two spots in the internal control (IC) and test (T) area. Negative samples developed a single spot in IC area alone.

### Statistical Analysis

Data were analyzed and plotted using SigmaPlot 11.0 or graph pad prism version 5.0 software. Cut-off values for each diagnostics were defined as the corresponding Mean+2SD calculated from the sera of normal healthy controls. Sensitivity was defined as the percentage of the laboratory-confirmed cases of leptospirosis whose serum samples gave mean OD greater than the relevant cutoff value. Specificity was calculated as the percentage of the control individuals whose samples gave mean OD below the relevant cut-off value. The positive and negative predictive values (PPV and NPV respectively) are the proportions of true positive and true negative results. The percentage agreement between different diagnostics and MAT, and the corresponding kappa coefficients, were determined using Epi Info version 6.0 (Centers for Disease Control and Prevention, Atlanta, GA).

## Results

### Seroprevalence of Leptospiral Infection

Circulating anti-leptospiral antibodies were detected by MAT using a panel of 12 leptospiral serogroups. Five serogroups were found to be highly prevalent: Australis (27.5%), Ballum (25.8%), Grippotyphosa (12.5%), Autumnalis (11.7%), and Pomona (10%). The highest antibody titre of 1:2560 was observed for Australis and Autumnalis. The prevalent serogroups with their MAT titre are given in S2 Table. The sera samples that were positive for Australis (1.3%) and Autumnalis (1.4%) showed cross-reactivity to the serogroup Ballum.

### SDS-PAGE and Immunoblot Analysis

**Fig 1:**
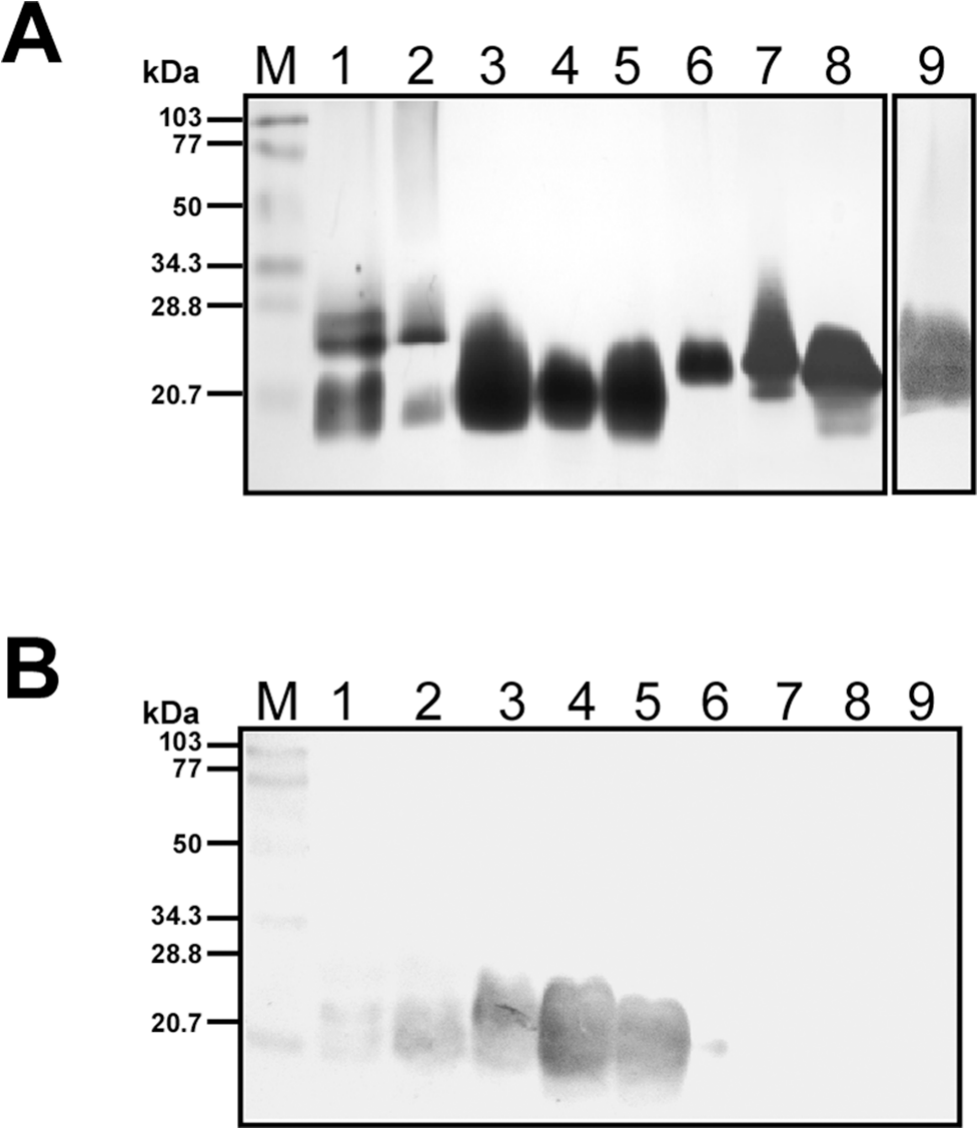
SDS PAGE profile and the immunoreactivity of the extracted LPS. **A** SDS PAGE profile of purified LPS (100 μg) detected by silver staining. Lane M- BioRad low range molecular weight protein marker, LPS from Autumnalis (Lane 1), Australis (Lane 2), Ballum (Lane 3), Grippotyphosa (Lane 4), Pomona (Lane 5), Andamana (Lane 6), *E*. *coli* (Lane 7), *Serratia marcescens* (Lane 8), and *Citrobacter freundi* (Lane 9). **B** Western blot of the purified LPS (50 μg) with pooled patients’ sera. Lane M- BioRad low range molecular weight protein marker, LPS from Autumnalis (Lane 1), Australis (Lane 2), Ballum (Lane 3), Grippotyphosa (Lane 4), Pomona (Lane 5), Andamana (Lane 6), *E*. *coli* (Lane 7), *Serratia marcescens* (Lane 8), and *Citrobacter freundi* (Lane 9).

Extracted LPS were used as antigens in all diagnostic formats. The prepared antigen contained ~575–625 μg/mL of LPS and no detectable levels of protein contaminations. The apparent molecular masses of extracted LPS were in the range of 14–27 kDa as determined by SDS-PAGE and silver staining (Fig 1A). Immunoblots developed with pooled patients’ sera showed reactivity with LPS from pathogenic serogroups including Autumnalis, Australis, Ballum, Grippotyphosa, and Pomona. No reactivity was observed for LPS from non-pathogenic serogroup Andamana, and other bacterial LPS (Fig 1B). To confirm the serogroup specificity of the extracted LPS, the blots were probed with homologous patients’ sera. Serogroup specific reactivity was observed for all leptospiral LPS against their homologous sera (S2 Fig) confirming the serogroup specificity.

### Sensitivity and Specificity of LPS Based IgM ELISA

**Fig 2:**
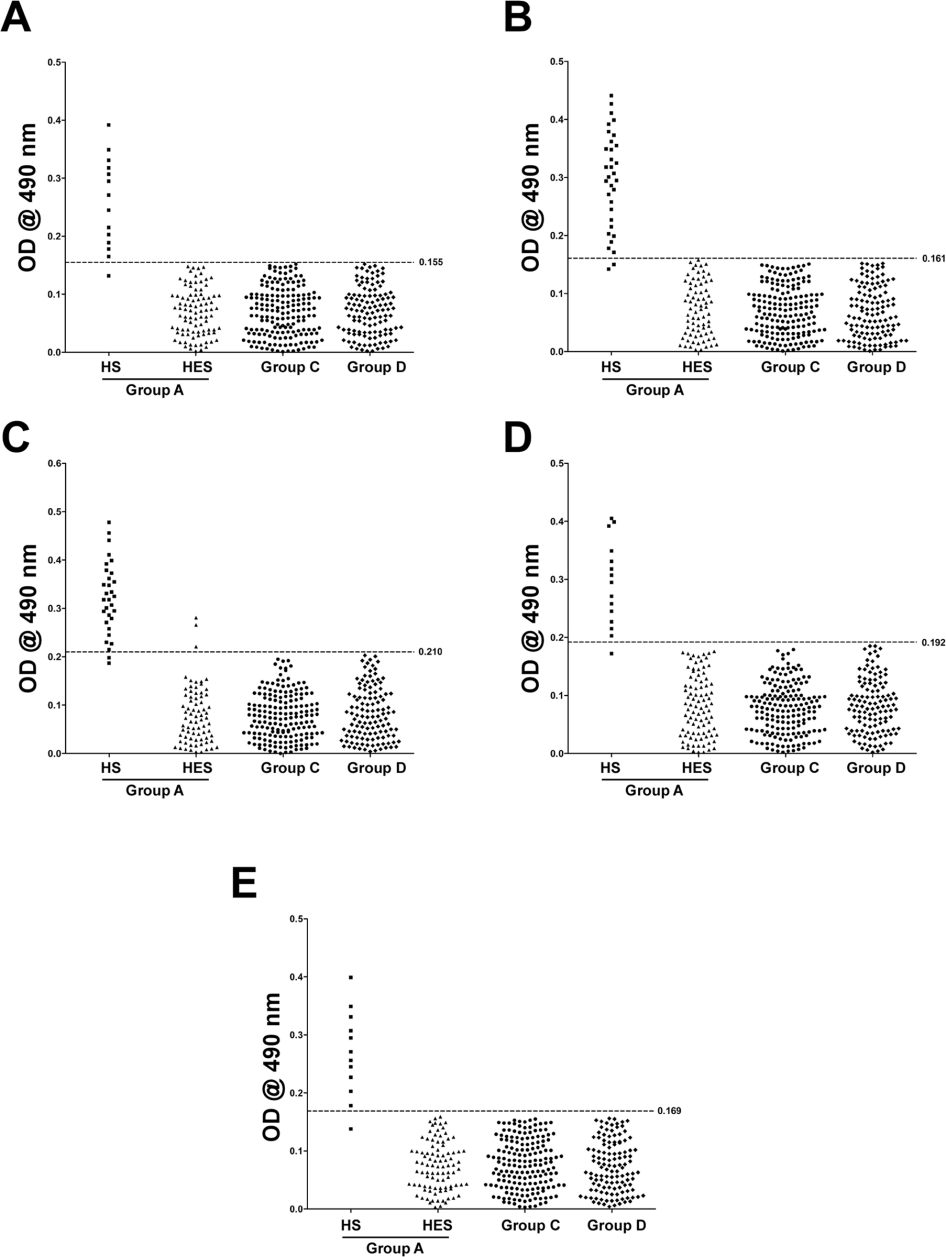
Evaluation of LPS based IgM-ELISAs. Study groups are indicated on the *x* axis and the optical density (OD) at 490 nm on the *y* axis. IgM responses to various leptospiral LPS: Autumnalis (A), Australis (B), Ballum (C), Grippotyphosa (D), Pomona (E) are shown. Study groups were as described in Table 1. The dashed line represents the cut-off values for each antigens with the absolute cut-off values on the right.

The overall results of the LPS based IgM ELISA are given in Fig 2. The mean + 2 SD absorbance values for seronegative healthy individuals were defined as the cut off values to achieve diagnostic specificity of the ELISAs in comparison with MAT. The cut-off values for IgM ELISA were 0.155, 0.161, 0.210, 0.192 and 0.169 for Autumnalis, Australis, Ballum, Grippotyphosa and Pomona respectively. The IgM ELISA demonstrated sensitivity of 92.9%, 93.9%, 93.6%, 93.3% and 91.7% for homologous sera of Autumnalis, Australis, Ballum, Grippotyphosa and Pomona respectively (S3 Table). Except for Ballum LPS that showed 4.05% cross-reactivity for heterologous sera, other LPS showed no cross reactivity.

### Sensitivity and Specificity of LPS Based Dot Blot Assay

**Fig 3:**
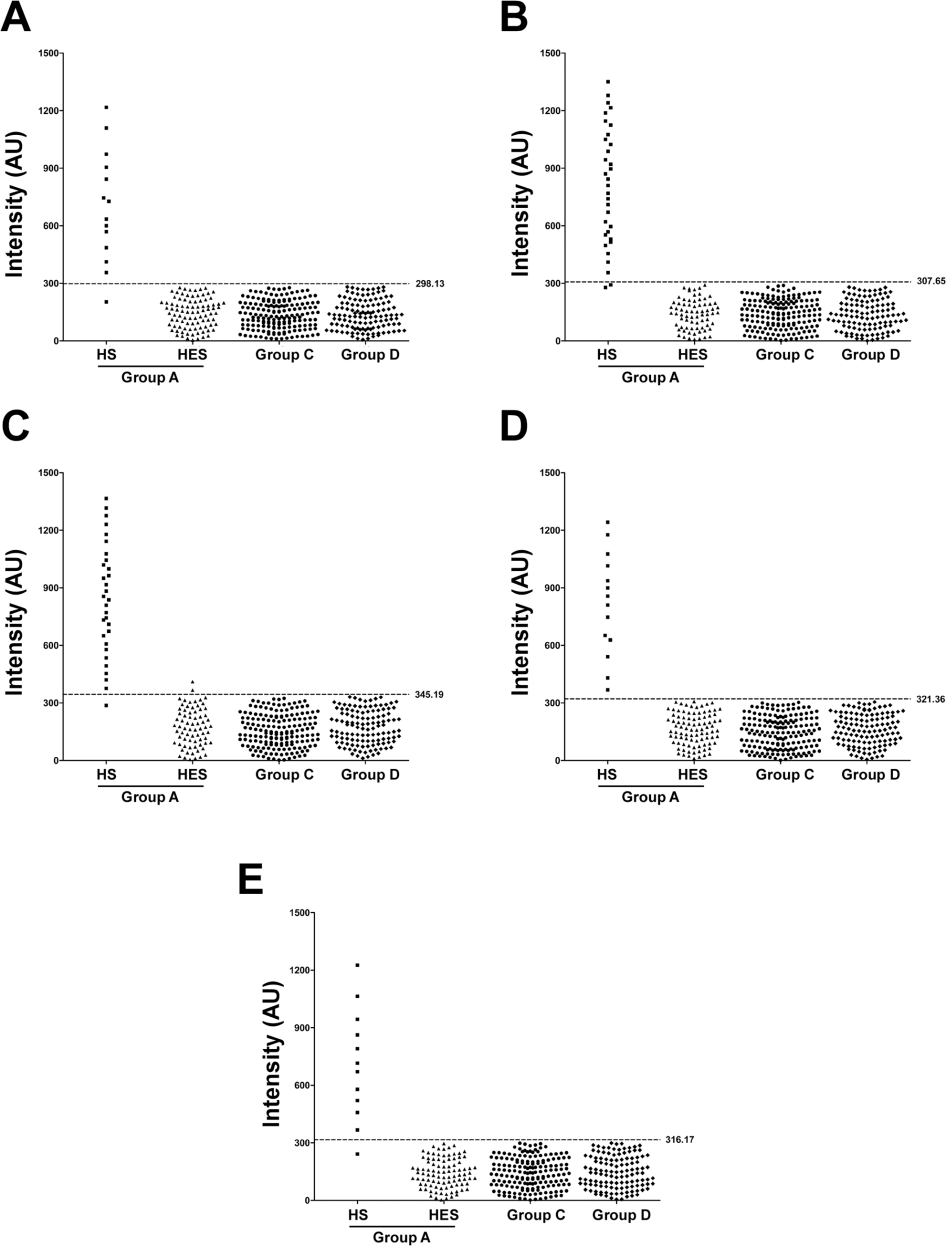
Evaluation of LPS based IgM-dot blot assay. Study groups are indicated on the *x* axis and densitometric values expressed in arbitrary units (AU) on the *y* axis. IgM responses to various leptospiral LPS: Autumnalis (A), Australis (B), Ballum (C), Grippotyphosa (D), Pomona (E) are shown. The dashed line represents the cut-off values for each antigens with the absolute cut-off values on the right.

The overall sensitivity and specificity of IgM dot blot assay developed with leptospiral LPS are shown in Fig 3. The mean + 2 SD of the AU values from seronegative healthy individuals were defined as the cutoff values to achieve diagnostic sensitivity and specificity. The dot blot with various LPS demonstrated increased sensitivity in the range of ~91 to 100% for homologous sera (S4 Table). Similar to IgM- ELISA cross reactivity (4.05%) was observed with Ballum LPS based dot blot.

### Sensitivity and Specificity of LPS Based LFA

**Fig 4:**
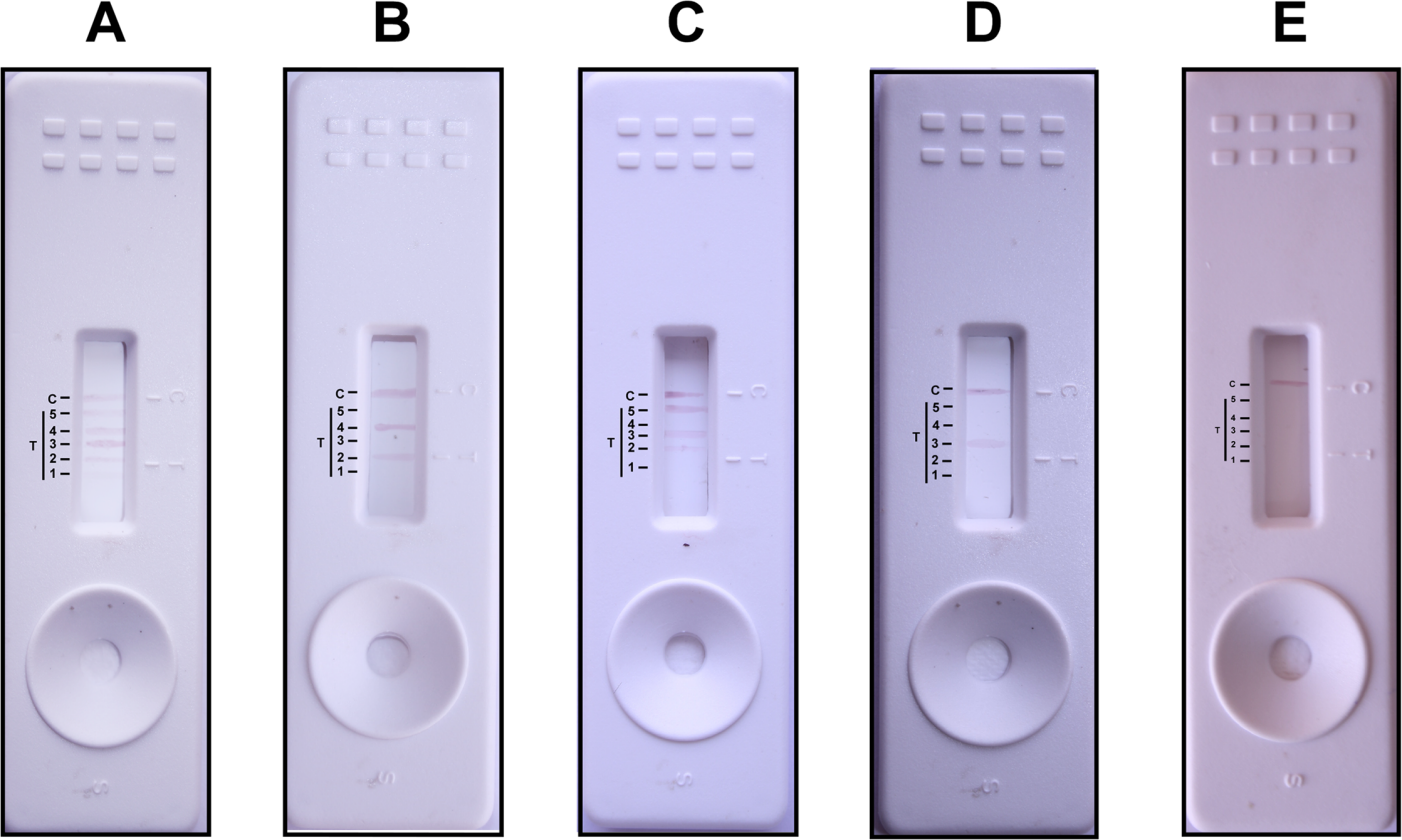
Evaluation of ICG-based LFA using patients’ sera samples. The extracted LPS from Autumnalis (1), Australis (2), Ballum (3), Grippotyphosa (4), Pomona (5) and anti-human IgM (C) were spotted as line at equal distance in the test and control areas as depicted in the figure and antibody was detected from pooled patients’ sera positive for five serogroups (A), pooled sera positive for Australis and Grippotyphosa (B), pooled sera positive for Australis, Ballum and Pomona (C), pooled sera positive for Ballum (D), and pooled sero- negative healthy controls (E).

The representative results of ICG based LFA are shown in Fig 4. The sensitivity of LFA was shown to be in the range of ~93 to 100% for homologous sera (Table 2 and S3 Fig). The ICG-LFA developed was able to detect anti-leptospiral antibodies in patients’ sera and can discriminate it from other bacterial infections (S3 Fig) making its applicability as a genus specific test. As seen for other serological assays, except for Ballum LPS (cross-reactivity of 1.1%) other LPS showed no cross-reactivity for heterologous sera. The Wilcoxon analysis showed that the ICG based LFA developed with leptospiral LPS was not significantly different from the gold standard MAT (P > 0.05). The developed ICG-LFA had PPV in the range of 85–100% and NPV >90%. Hence, the predictive values provided useful information about the utility of the test for efficient diagnosis of leptospirosis. The ICG-LFA with high PPV almost confirms the diagnosis during the first week of the disease further strengthening our claim of using the developed ICG-LFA for early sergroup specific diagnosis of leptospirosis (Table 2). The agreement between ICG-LFA and the standard criteria for diagnosis (MAT) was significant, as indicated by a κ value greater than 0.4.

**Table 2:** Sensitivity, Specificity, PPV, NPV and kappa value for LPS based ICG-LFA.

Antigen (LPS)	Number of samples included	Sensitivity (%)	Specificity (%)	PPV (%)	NPV (%)	kappa value
Autumnalis	14	92.86	100	100	99.74	0.962
Australis	33	93.93	99.46	93.93	99.46	0.934
Ballum	31	93.55	99.19	90.63	99.46	0.914
Grippotyphosa	15	93.33	100	100	99.74	0.964
Pomona	12	100	99.48	85.71	100	0.921

## Discussion

LPS is the major antigenic component present in the outer membrane of leptospires and considered best for diagnosis as they are serogroup specific [20]. Current laboratory diagnosis of leptospirosis mainly relies on serological methods, and the most widely used reference method is the serogroup specific MAT. Though MAT detects both anti-leptospiral IgM and IgG, most of the agglutinating antibodies encountered in MAT are IgM that are produced against the LPS during the early phase of illness [21, 22]. MAT forms the bases for serological diagnosis and classification of leptospirosis. Generally MAT is performed with locally predominant leptosprial serovars as live antigens. Although MAT is considered as gold standard test, it is complex to control, perform, and interpret. Live cultures of at least the locally predominant serovars are required to be maintained for its use as antigens in diagnosis. Apart from these drawbacks MAT suffers from major issues including (i) continuous risk of cross-contamination of the antigen cultures, (ii) MAT titers being affected by the culture medium and (iii) repeated subculturing of large numbers of leptospiral strains are hazardous for laboratory workers [23]. Moreover, in an outbreak situation examination of such large number of serum samples by a complex technique like MAT may compromise the quality of results.

Under sudden upsurge conditions, the use of an alternative rapid serological diagnostic test is highly recommended. Numerous alternative techniques have been described and evaluated till date. Serological tests including the microcapsule agglutination test (MCAT), Lepto Dipstick, Lepto Lateral Flow, and Lepto Dri Dot have been evaluated as rapid tests for leptospirosis. But they have major drawbacks including low sensitivity during the acute stage of the disease and are mostly genus specific [24–27]. The tests being evaluated or commercialized use crude antigenic preparations abundant in leptospiral proteins that are highly conserved between different serogroups. Thus leptospiral LPS that are serogroup specific and reported as the first antigen to be recognized as PAMP (Pathogen Associated Molecular Patterns) by the host immune cells can be employed for rapid and serogroup specific diagnosis of acute leptospirosis.

So far MAT doesn’t have an alternative reproducible serogroup specific test and hence LPS based diagnostics in the form of ELISA /dot blot /ICG-LFA was evaluated in the present study. Leptospiral LPS was extracted from the 5 predominant locally circulating serogroups and tested for its diagnostic sensitivity and specificity. The diagnostic specificity of LPS based ELISA /dot blot was found to be ~ 95%. Except for Ballum LPS, no cross reactivity was observed for other LPS, when tested for heterologous sera. The cross reactivity may be due to the antigenic structural similarities of the Ballum LPS with Autumnalis / Australis that needs to validated in detail in future studies.

Further to improvise the rapidity of the technique and make it applicable in outbreak situations, gold nano conjugated ICG-LFA was developed. Several ICG based detection of leptospiral antibodies in clinical samples have been evaluated [26] and reported to have low sensitivities during early phase of the disease. In a study, monoclonal antibody, 1H6 was produced and evaluated in ICG based diagnostics to detect leptospiral antigens in urine. The produced antibody reacted with a 12 kDa leptospiral LPS. The overall sensitivity and specificity was found to be <90% during the early stage of illness. The developed ICG method was able to detect *Leptospira* antigen and was not able to identify the infecting serogroup of *Leptospira* [9]. Therefore we tried to develop ICG-LFA based diagnosis with leptospiral LPS and to detect IgM antibodies in human sera. The present ICG-LFA with an array of leptospiral LPS may be an ideal test system to design a rapid, serogroup specific diagnosis of leptospirosis. LFA for rapid diagnosis using heat resistant antigens has been evaluated for field application in an endemic region [27] with a sensitivity of 34.3% during 2–4 days of illness. But the use of purified LPS in an ICG-based diagnostic format has appreciable sensitivity and specificity. Moreover the test was practiced as an array incorporating LPS from different pathogenic serogroups as a single strip and therefore in one application we may be able to find out the infecting serogroup without compromising the sensitivity. The application of single array of LPS for serogroup specific diagnosis is first of its kind. This assay could potentially be evaluated and applied as an alternate for MAT to identify the locally predominant serogroups in endemic regions like India.

In conclusion, the confirmatory diagnosis of leptospirosis by MAT is only feasible in reference laboratories. Thus in developing countries particularly South East Asian countries like India, access to reference serodiagnostic MAT may be difficult and limited resources could preclude the inclusion of resources for the performance of such complex assays. Therefore the developed simple, rapid serogroup specific LPS based ICG-LFA may be a productive assay for the diagnosis of leptospirosis in the early stages of illness where timely initiation of antibiotics may be critical [28].

## Supporting Information

S1 Fig Determination of optimal antigen (LPS) concentration for IgM Dot blot assay. Various concentrations of LPS from *L*. *interrogans* serovar Autumnalis was probed with MAT positive sera for serovar Autumnalis.
(PDF)

S2 Fig Serogroup specific reactivity of extracted LPS. The extracted LPS from Autumnalis (Lane 1), Australis (Lane 2), Ballum (Lane 3), Grippotyphosa (Lane 4), Pomona (Lane 5), Andamana (Lane 6), *E*. *coli* (Lane 7), *Serratia marcescens* (Lane 8), and *Citrobacter freundi* (Lane 9) were separated on SDS PAGE and probed with homologous sera specific for Autumnalis (**A**), Australis (**B**), Ballum (**C**), Grippotyphosa (**D**), Pomona (**E**), and seronegative healthy controls (**F**). Lane M- BioRad low range molecular weight protein marker.
(PDF)

S3 Fig Evaluation of LPS based ICG-LFA. Study groups are indicated on the *x* axis and the optical density (OD) at 490 nm on the *y* axis. IgM responses to various leptospiral LPS: Autumnalis (A), Australis (B), Ballum (C), Grippotyphosa (D), Pomona (E) are shown. The dashed line represents the cut-off values for each antigens with the absolute cut-off values on the right. Study groups were as described in Table 1.
(PDF)

S1 Table Comparison of MAT with IgM ELISA/culture positivity. (PDF)

S2 Table Median MAT titers of 120 MAT positive sera sample. * indicates serogroup obtained by isolation was identical to the serogroup identified by MAT. Number of isolates obtained in the corresponding serogroups are given.
(PDF)

S3 Table Sensitivity, Specificity, PPV, NPV and kappa value of various leptospiral LPS based IgM ELISA against homologous sera. (PDF)

S4 Table Sensitivity, Specificity, PPV, NPV and kappa value of various leptospiral LPS based IgM dot blot against homologous sera. (PDF)
