# Supplementary material for: Lipopolysaccharide Specific Immunochromatography Based Lateral Flow Assay for Serogroup Specific Diagnosis of Leptospirosis in India
Source: PLoS One. 2015 Sep 4;10(9):e0137130. doi: 10.1371/journal.pone.0137130 (PMC4560487; doi:10.1371/journal.pone.0137130)
Supplement: S1 Fig — Various concentrations of LPS from L. interrogans serovar Autumnalis was probed with MAT positive sera for serovar Autumnalis. (PDF) [file pone.0137130.s001.pdf]

S1 Fig: Determination of optimal antigen (LPS) concentration for IgM Dot blot assay

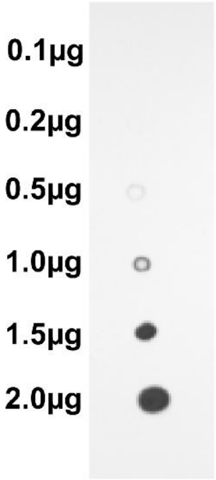

Various concentrations of LPS from *L. interrogans* serovar Autumnalis was probed with MAT positive sera for serovar Autumnalis
