# Supplementary material for: Lipopolysaccharide Specific Immunochromatography Based Lateral Flow Assay for Serogroup Specific Diagnosis of Leptospirosis in India
Source: PLoS One. 2015 Sep 4;10(9):e0137130. doi: 10.1371/journal.pone.0137130 (PMC4560487; doi:10.1371/journal.pone.0137130)
Supplement: S2 Fig — The extracted LPS from Autumnalis (Lane 1), Australis (Lane 2), Ballum (Lane 3), Grippotyphosa (Lane 4), Pomona (Lane 5), Andamana (Lane 6), E. coli (Lane 7), Serratia marcescens (Lane 8), and Citrobacter freundi (Lane 9) were separated on SDS PAGE and probed with homologous sera specific for Autumnalis (A), Australis (B), Ballum (C), Grippotyphosa (D), Pomona (E), and seronegative healthy controls (F). Lane M- BioRad low range molecular weight protein marker. (PDF) [file pone.0137130.s002.pdf]

S2 Fig: Serogroup specific reactivity of extracted LPS

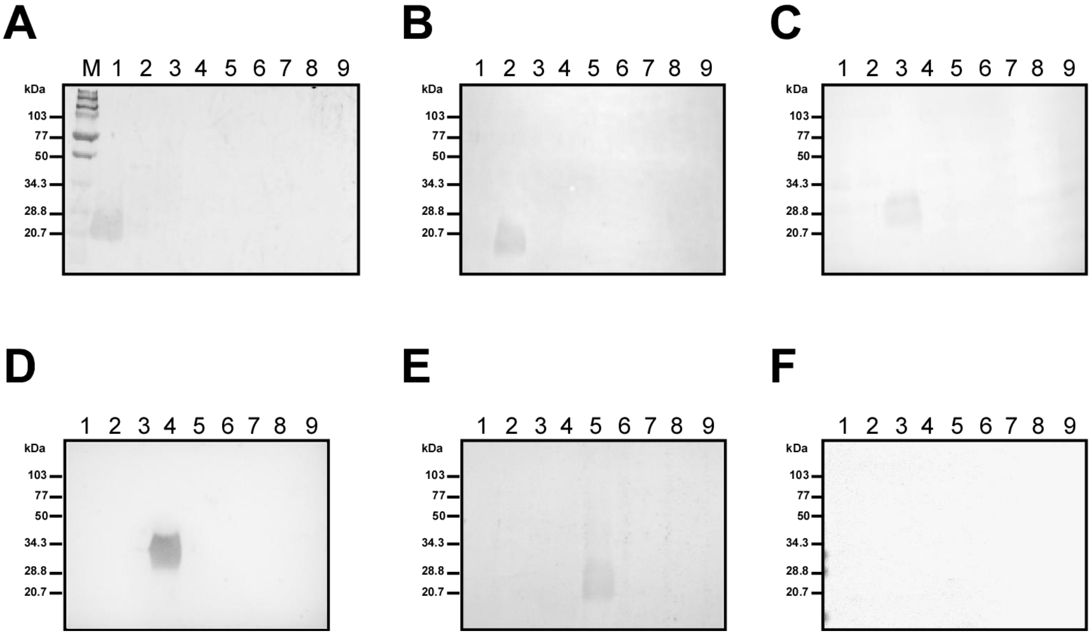

The extracted LPS from Autumnalis (Lane 1), Australis (Lane 2), Ballum (Lane 3), Grippotyphosa (Lane 4), Pomona (Lane 5), Andamana (Lane 6), *E. coli* (Lane 7), *Serratia marcescens* (Lane 8), and *Citrobacter freundi* (Lane 9) were separated on SDS PAGE and probed with homologous sera specific for Autumnalis (**A**), Australis (**B**), Ballum (**C**), Grippotyphosa (**D**), Pomona (**E**), and seronegative healthy controls (**F**). Lane M- BioRad low range molecular weight protein marker.
