# Supplementary material for: Lipopolysaccharide Specific Immunochromatography Based Lateral Flow Assay for Serogroup Specific Diagnosis of Leptospirosis in India
Source: PLoS One. 2015 Sep 4;10(9):e0137130. doi: 10.1371/journal.pone.0137130 (PMC4560487; doi:10.1371/journal.pone.0137130)
Supplement: S3 Fig — Study groups are indicated on the x axis and the optical density (OD) at 490 nm on the y axis. IgM responses to various leptospiral LPS: Autumnalis (A), Australis (B), Ballum (C), Grippotyphosa (D), Pomona (E) are shown. The dashed line represents the cut-off values for each antigens with the absolute cut-off values on the right. Study groups were as described in Table 1. (PDF) [file pone.0137130.s003.pdf]

S3 Fig: Evaluation of LPS based ICG-LFA

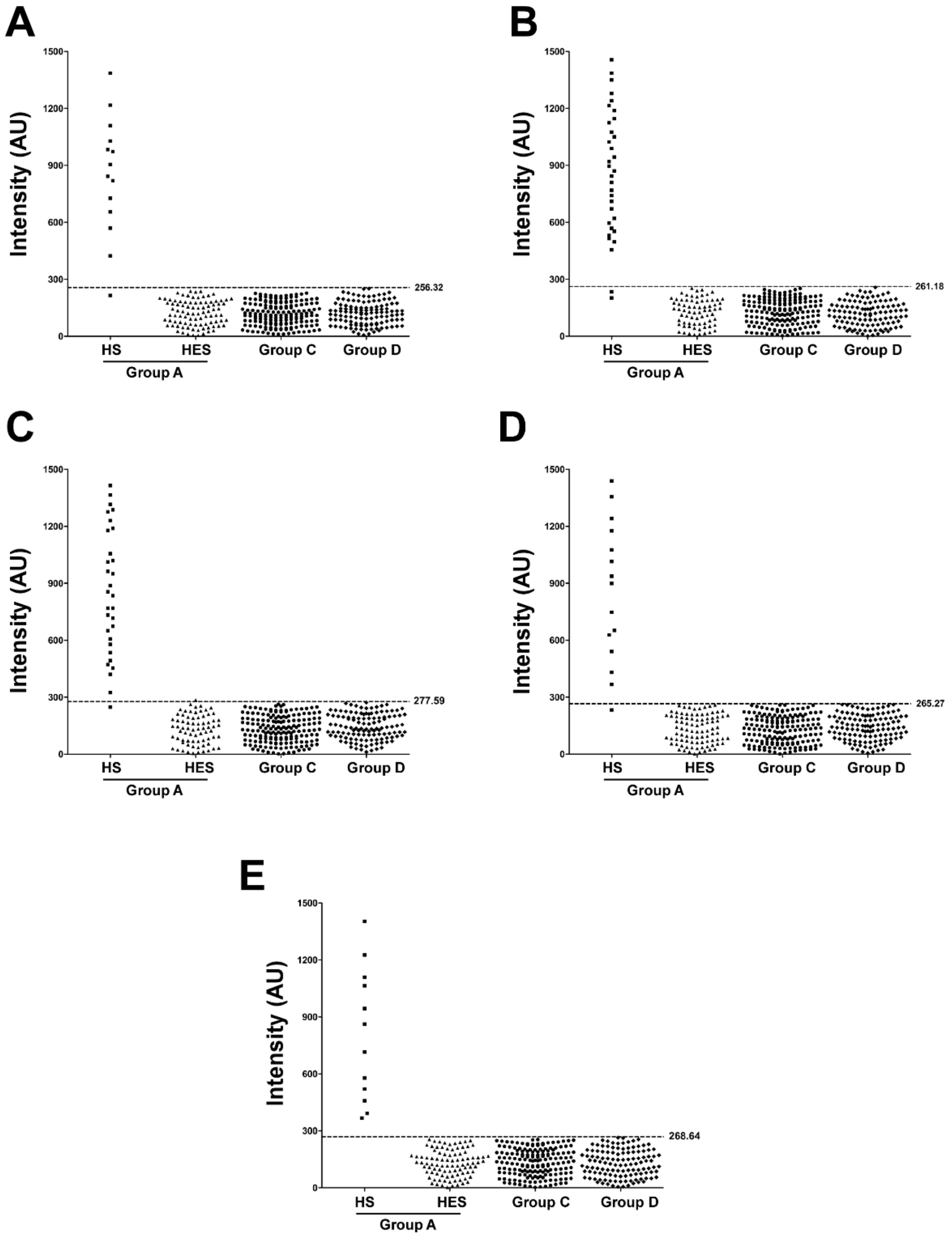

Study groups are indicated on the *x* axis and the optical density (OD) at 490 nm on the *y* axis. IgM responses to various leptospiral LPS: Autumnalis (A), Australis (B), Ballum (C), Grippotyphosa (D), Pomona (E) are shown. The dashed line represents the cut-off values for each antigens with the absolute cut-off values on the right.Study groups were as described in Table 1.
