# Supplementary material for: Lipopolysaccharide Specific Immunochromatography Based Lateral Flow Assay for Serogroup Specific Diagnosis of Leptospirosis in India
Source: PLoS One. 2015 Sep 4;10(9):e0137130. doi: 10.1371/journal.pone.0137130 (PMC4560487; doi:10.1371/journal.pone.0137130)
Supplement: S1 Table — (PDF) [file pone.0137130.s004.pdf]

S1 Table: Comparison of MAT with IgM ELISA/culture positivity

MAT	IgM ELISA		Total	MAT	Culture positivity		Total
	Positive	Negative			Positive	Negative	
Positive	90	30	120	Positive	7	113	120
Negative	40	40	80	Negative	2	78	80
Total	130	70	200	Total	9	191	200
Sensitivity: 75%<br>Specificity : 50%				Sensitivity: 5.8%<br>Specificity : 97.5%			
