# Supplementary material for: Lipopolysaccharide Specific Immunochromatography Based Lateral Flow Assay for Serogroup Specific Diagnosis of Leptospirosis in India
Source: PLoS One. 2015 Sep 4;10(9):e0137130. doi: 10.1371/journal.pone.0137130 (PMC4560487; doi:10.1371/journal.pone.0137130)
Supplement: S2 Table — * indicates serogroup obtained by isolation was identical to the serogroup identified by MAT. Number of isolates obtained in the corresponding serogroups are given. (PDF) [file pone.0137130.s005.pdf]

**S2 Table: Median MAT titers of the 120 MAT positive sera samples**

| Serogroup | Number positive n (%) | MAT titer | Number of isolates obtained (n = 9)* |
|---|---|---|---|
| Autumnalis | 14 (11.7) | 1:2560 | 2 |
| Australis | 33 (27.5) | 1:2560 | 1 |
| Ballum | 31 (25.8) | 1:640 | - |
| Grippotyphosa | 15 (12.5) | 1:640 | - |
| Pomona | 12 (10) | 1:640 | 1 |
| Canicola | 8 (6.7) | 1:160 | 4 |
| Icterohaemorrhagiae | 4 (3.3) | 1:160 | - |
| Javanica | 3 (2.5) | 1:160 | 1 |

* Indicate serogroup obtained by isolation was identical to the serogroup identified by MAT. Number of isolates obtained in the corresponding serogroups are given.
