# Supplementary material for: Lipopolysaccharide Specific Immunochromatography Based Lateral Flow Assay for Serogroup Specific Diagnosis of Leptospirosis in India
Source: PLoS One. 2015 Sep 4;10(9):e0137130. doi: 10.1371/journal.pone.0137130 (PMC4560487; doi:10.1371/journal.pone.0137130)
Supplement: S3 Table — (PDF) [file pone.0137130.s006.pdf]

S3 Table: Sensitivity, Specificity, PPV, NPV and kappa value of various leptospiral LPS based IgM ELISA against homologous sera

Antigen (LPS)	Sensitivity %	Specificity %	PPV %	NPV %	kappa value
Autumnalis	92.86	99.74	92.86	99.74	0.926
Australis	93.93	99.46	93.93	99.46	0.934
Ballum	93.55	99.19	90.63	99.46	0.914
Grippotyphosa	93.33	99.74	93.33	99.74	0.931
Pomona	91.67	99.74	91.67	99.74	0.914
