# Supplementary material for: Lipopolysaccharide Specific Immunochromatography Based Lateral Flow Assay for Serogroup Specific Diagnosis of Leptospirosis in India
Source: PLoS One. 2015 Sep 4;10(9):e0137130. doi: 10.1371/journal.pone.0137130 (PMC4560487; doi:10.1371/journal.pone.0137130)
Supplement: S4 Table — (PDF) [file pone.0137130.s007.pdf]

S4 Table: Sensitivity, Specificity, PPV, NPV and kappa value of various leptospiral LPS based IgM dot blot against homologous sera

Antigen (LPS)	Sensitivity %	Specificity %	PPV %	NPV %	kappa value
Autumnalis	92.86	99.74	92.86	99.74	0.926
Australis	93.93	99.73	96.88	99.46	0.950
Ballum	96.77	99.19	90.91	99.73	0.932
Grippotyphosa	100	99.48	87.5	100	0.931
Pomona	91.67	99.74	91.67	99.74	0.914
